# Assessing the performance of ChatGPT-4 and ChatGPT-4o in lung cancer diagnoses

**DOI:** 10.1186/s12967-025-06337-1

**Published:** 2025-03-18

**Authors:** Jinru Yang, Xing Cai, Xiaofang Dai, Conghua Xie

**Affiliations:** 1 Department of Pulmonary Oncology, Hubei Key Laboratory of Tumor Biological Behaviors, Hubei Cancer Clinical Study Center, Zhongnan Hospital of Wuhan University, Wuhan, 430071 Hubei China; 2 Cancer Center, Union Hospital, Tongji Medical College, Huazhong University of Science and Technology, Wuhan, 430022 Hubei China; 3 Wuhan Research Center for Infectious Diseases and Cancer, Chinese Academy of Medical Sciences, Wuhan, 420071 Hubei China

**Keywords:** Artificial intelligence, Lung cancer, Diagnosis

To the Editor,

Lung cancer is a highly invasive and prevalent disease, which is a leading cause of cancer death globally [1]. Timely diagnosis is key to improving outcomes. Pulmonary CT is essential in detecting lung cancer, relying on signs like lobulation, spiculation, pleural indentation, and vacuolar sign, which help experts assess the type, location, and progression of the disease, aiding in clinical decision-making. Recently, with the rapid development of artificial intelligence (AI) technology, the large language models (LLM) such as ChatGPT-4o, ChatGPT-4, and Google Bard introduced image-reading capabilities [2], have offered new solutions for early lung cancer diagnosis [3], particularly in low- and middle-income regions with limited clinical expertise, which can have significant impacts on cost-effectiveness and local healthcare resource allocation. Therefore, this study evaluates the accuracy and cost-effectiveness of ChatGPT versus clinical physicians in diagnosing lung cancer, using published cases.

This study, conducted in January 2025, reviewed 60 lung cancer cases. We extracted medical history, images (CT, H&E, IHC, PET/CT, etc.), and multiple-choice options from the cases to create 60 question documents. These were entered into ChatGPT-4 and 4o, prompting the models to provide the most likely and second most likely answers, along with confidence ratings. Two chief lung oncologists independently conducted blinded evaluations for comparison. For cost analysis, we used the 2023 Eurozone average labor cost (35.6 EUR/hour or 38.7 USD/hour).

After evaluating responses from ChatGPT-4o, ChatGPT-4 and two physicians, results showed that for the top diagnosis, ChatGPT-4o (73.33%) was comparable to ChatGPT-4 (60.00%) and physician-2 (88.67%) (*P* = 0.121, *P* = 0.068), but significantly lower than physician-1 (95.00%) (*P* = 0.001). For the top two diagnoses, ChatGPT-4o (86.67%) also showed no significant difference from ChatGPT-4 (73.33%) and physician-2 (95.00%), but was significantly lower than physician-1 (98.33%) (*P* = 0.015). ChatGPT-4o had higher confidence in its first diagnosis compared to ChatGPT-4 (*P* < 0.001), but lower than both physicians (*P* < 0.001). Confidence for the second diagnosis dropped but remained higher than ChatGPT-4 (*P* < 0.001), with no significant difference from the physicians. ChatGPT-4o and ChatGPT-4 had significantly lower time and cost (*P* < 0.001) compared to the doctors, with ChatGPT-4o being the fastest and most cost-effective (Fig. 1).

Our research shows that ChatGPT-4o demonstrates high accuracy in lung cancer diagnosis, nearing the performance of clinical doctors, with clear advantages in time and cost. While ChatGPT-4 struggles with longer inputs and multimodal data, ChatGPT-4o’s capabilities make it a strong tool for initial lung cancer diagnosis, especially in regions with limited medical expertise. ChatGPT-4o also maintains consistent performance, even in multitasking or emergency situations where physician accuracy might decrease. However, the study’s small sample size and limited representation of lung tumor variability are notable limitations. Larger studies and more AI models are needed for further validation. Despite these limitations, our study suggests ChatGPT-4o could serve as a low-cost, rapid diagnostic tool, aiding doctors in improving diagnostic accuracy and providing valuable guidance for non-medical professionals. It lays a foundation for future AI-assisted lung cancer diagnosis and early intervention.

**Fig. 1:**
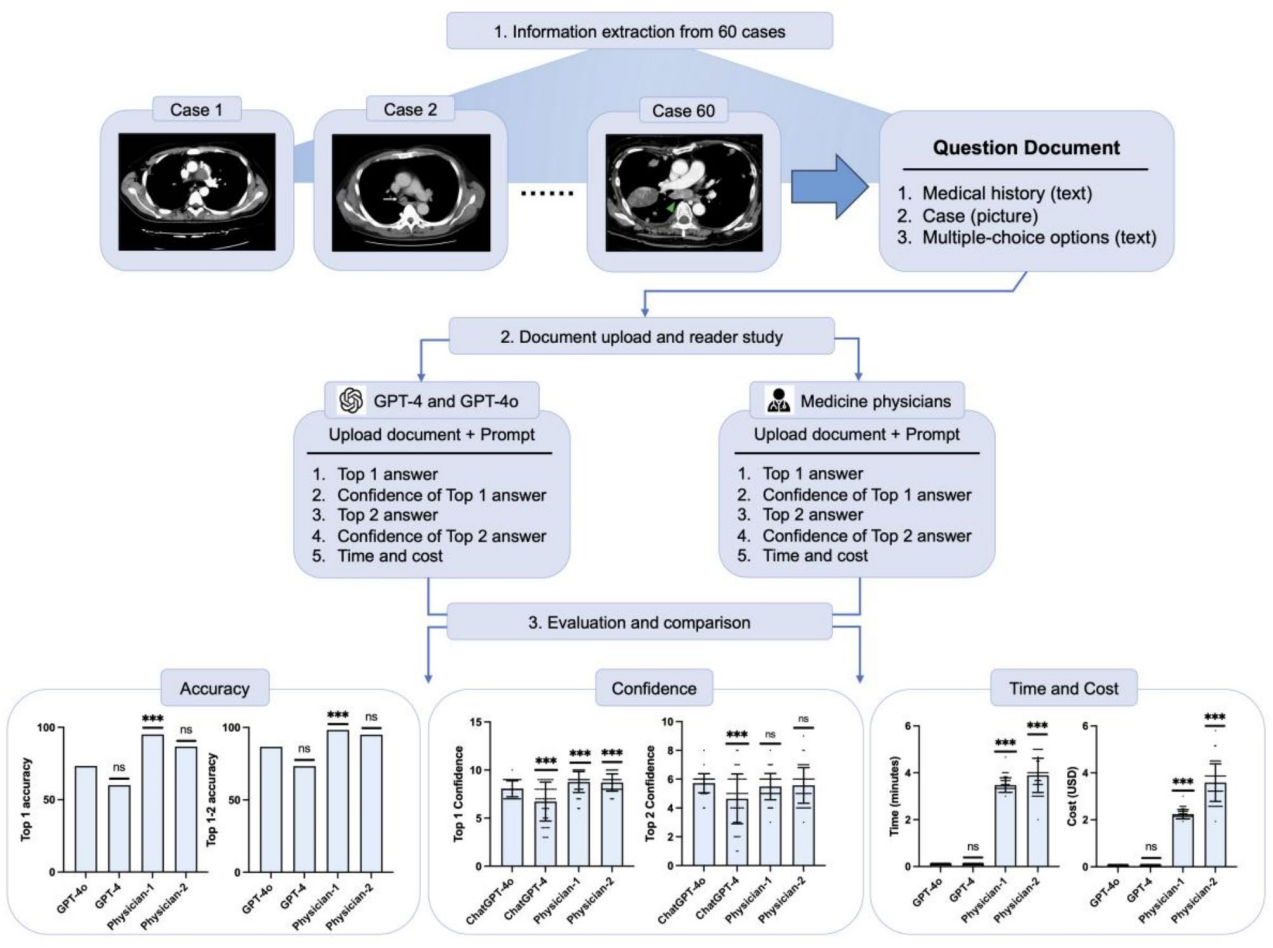
Workflow and results comparing the accuracy, confidence, time, and cost of ChatGPT-4, ChatGPT-4o and physicians in diagnosing lung cancer. ChatGPT-4o served as the control. Accuracy comparisons used Pearson’s chi-squared test, while confidence, time, and cost were compared with independent t-tests. ***: statistically significant; ns: not significant

## Electronic supplementary material

Below is the link to the electronic supplementary material.

Supplementary Material 1

## Data Availability

Publicly available data were analyzed in this study.

## Abbreviations

AI: Artificial intelligence
LLM: Large language models
ChatGPT: Chat generative pretrained transformer
CT: Computed tomography
H&E: Hematoxylin and eosin
IHC: Immunohistochemistry
PET/CT: Positron emission tomography/computed tomography
